# Comparison of growth characteristics between skeletal muscle satellite cell lines from diploid and triploid olive flounder *Paralichthys olivaceus*

**DOI:** 10.7717/peerj.1519

**Published:** 2016-01-05

**Authors:** Li-min Peng, Yuan Zheng, Feng You, Zhi-hao Wu, Xungang Tan, Shuang Jiao, Pei-jun Zhang

**Affiliations:** 1 Key Laboratory of Experimental Marine Biology, Institute of Oceanology, Chinese Academy of Sciences, Qingdao, China; 2 Laboratory for Marine Biology and Biotechnology, Qingdao National Laboratory for Marine Science and Technology, Qingdao, China; 3 University of Chinese Academy of Sciences, Beijing, China

**Keywords:** Olive flounder *Paralichthys olivaceus*, Muscle satellite cell line, Differentiation, Transfection, Diploid and triploid

## Abstract

Objectives. According to myosatellite cell lines (MSCs) established *in vitro* from diploid and triploid flounder, we compared the characters of growth and differentiation of their MSCs. The results would be useful for learning the muscle development mechanism in teleosts.

Materials and Methods. The skeletal muscle cells from the diploid and triploid olive flounder *Paralichthys olivaceus* were isolated and cultured *in vitro*, respectively, and the cells were characterized at the morphology and molecular level; meanwhile, the performance of these cells’ proliferation and differentiation were analyzed.

Results. Two new skeletal muscle cell lines (POMSC_S(2n)_ and POMSC_S(3n)_) from diploid and triploid flounder have been respectively subcultured for 67 times and 66 times. The cultured cells were mostly spindle-like mononuclear cells. They have normal flounder diploid karyotype (2n=48*t*) and triploid karyotype (3n=72*t*), respectively. Muscle satellite cell gene marker (*pax7b*) and myogenic cell protein marker (Desmin) were all expressed in cells of two cell lines. Both of the cells could differentiate into the large polynucleated muscle fibre cells, and immunofluorescence reactions of myosin heavy chain (MyHC) were positive. There were more cells of POMSC_S(3n)_ to differentiate into the muscle fibre cells than that of POMSC_S(2n)_. However, POMSC_S(2n)_ cells proliferated more rapidly than those of POMSC_S(3n)_ (*P* < 0.05). The significant fluorescent signals were observed in both POMSC_S(2n)_ and POMSC_S(3n)_ cells after transfected with pEGFP-N3 reporter plasmid.

Conclusions. The two cell lines have been established and characterized as MSCs. We suppose that it might be the differentiation capacity, rather than the proliferation activity of MSCs to play a key role in the better growth of triploid ones than diploid. Both cell lines will become the ideal tools to learn the mechanism of fish MSCs proliferation, differentiation and regeneration during muscle development in the future.

## Introduction

Fish muscle originated from skeletal muscle satellite cells (MSCs) is a very important tissue for fish swimming and live, and one of the food resources for human. Fish body weight mainly depends on both muscle fibre number increase (hyperplasia) and muscle fibre size increase (hypertrophy) as skeletal muscle contributes to 40–60% in fish body mass (Qin, Gu & Zhang, 2000). MSCs are a heterogeneous population of stem and progenitor cells originated from embryonic mesoderm, and their activation, proliferation and differentiation are the crucial basis for the muscle hyperplasic and hypertrophic growth. *In vitro*, cultured MSCs can realize a complete genesis process: proliferation, differentiation, fusion and the formation of the multinucleated myotubes (Fauconneau & Paboeuf, 2001a). The research on molecular mechanism of fish muscle satellite cell renewal, activation and etc. could increase the understanding of the skeletal muscle growth (Mommsen, 2001; Chapalamadugu et al., 2009). In teleost, the researches regarding muscle satellite cells have been carried out mainly in salmonids, cyprinids and sparidae, such as rainbow trout (*Oncorhynchus mykiss*) (Powell, Dodson & Cloud, 1989; Fauconneau & Paboeuf, 2000; Fauconneau & Paboeuf, 2001b; Gabillard, Sabin & Paboeuf, 2010), Atlantic salmon (*Salmo salar* L.) (Bower & Johnston, 2009), carp (*Cyprinus carpio*) (Koumans et al., 1990; Fauconneau & Paboeuf, 2001b), and gilthead sea bream (*Sparus aurata*) (Montserrat et al., 2007). An *in vitro* system for trout muscle satellite cell culture was established and used to examine the effect of *myostatin* (MSTN) on proliferation or differentiation of myogenic cells (Seiliez, Sabin & Gabillard, 2012). But compared with other vertebrates, the research on muscle satellite cells of fish is limited.

Growth rate is one of the paramount characteristics in fish commercial production. Triploid fish are expected to show a higher growth potential due to their sterility or reduced gonadal development. At present, induction of triploidy has been achieved in many fishes, such as carp, bighead carp (*Aristichthys mobilis*), large yellow croaker (*Larimichthys crocea*) and turbot (*Scophthatmus maximus*). Some papers showed that growth of triploid fishes could be more rapid than that of diploid ones (Cal et al., 2006; Maxime, 2008). Johnston (1999) found that muscle growth and development of triploid Atlantic salmon displayed a lower density of satellite cells, reduced rates of fibre recruitment, hypertrophy of muscle fibres, advanced development of myotubes, myofibrils and acetylcholinesterase staining at the myosepta (Tiwary, Kirubagaran & Ray, 2004). However, there were no further reports providing the characters data of triploid fish muscle cells, and there were almost no reports on culture of them.

Olive flounder *Paralichthys olivaceus* is one of the important mariculture fish species, which distributes in the coastal water of Japan, Korea and China. The previous studies on the molecular mechanism of muscle development mainly concerned the isolation and expression pattern analysis of muscle developmental related genes including *MyoD, myogenin*, and so on (Zhang et al., 2006b; Xu et al., 2007). The research on flounder MSC was only focused on the expression patterns of the transcription factors paired-box3 and 7 (*Pax3* and *Pax7*) homologues, MSC marker genes, during embryonic development (Jiao et al., 2015a; Jiao et al., 2015b). As a commercial fish, the induction conditions of triploid flounder were also studied (Kazoo, Shigeaki & Yoshihiro, 1989; You & Liu, 1995; Xu & Chen, 2010), and there was a report showing that the growth rate of triploidy was significantly higher than that of diploidy (Wang et al., 2011). The results of our further study demonstrated that the growth characters of triploidy in different development stages may be different (H Liu et al., 2015, unpublished data). So far, there were no reports on the mechanism of triploidy muscle development and MSC including triploid MSC in flounder. In this paper, two new muscle satellite cell lines (POMSC_S(2n)_ and POMSC_S(3n)_) respectively derived from the diploid and triploid flounder were established, and on the basis of analyzing their cytological features, the characteristics of these cell proliferation and differentiation were compared.

## Materials and Methods

### Fish

Adult flounders weighing about 100–200 g were purchased from Nanshan market (Qingdao, China), and were temporarily reared in a 500 L aerated seawater tank at the institute aquarium and fed with commercial particle food twice a day.

Triploid flounders were artificially induced according to You et al. (2001). Eggs extruded from female flounders with gently bulging bellies were fertilized with sperms, then cold shocked at 0–1 °C for 45 min at 5 min after fertilization to suppress the release of the second polar body. The embryos and fries were cultivated in a separate tank at 15–16°C. After checking their ploidy level by using flow cytometry with a PARTEC cell counter analyzer CCA-II (Partec, Münster, Germany), the viable juvenile triploidy were cultured under controlled conditions (photoperiod: 14/10 h (light/dark), aeration, filtered seawater) and fed with live bait or commercial particle food.

### Ethics statement

Experiments involving live animals were conducted in accordance with the “Regulations for the Administration of Affairs Concerning Experimental Animals” promulgated by the State Science and Technology Commission of Shandong Province. The study was approved by the ethics committee of Institute of Oceanology, Chinese Academy of Sciences.

### Tissue isolation and cell culture

A healthy live flounder weighing 123.5 g was selected to be anaesthetized by 40 mg ml^−1^ 3-Aminobenzoic acid ethyl ester methanesulfonate (MS-222, Yufubao, China) and wiped with 70% (v/v) ethanol. After removal of the skin and the red muscle tissue, 0.4–0.6 cm^3^ dorsal white muscle tissue was immediately excised and immersed in Minimum Essential Medium (MEM, Invitrogen, Waltham, MA, USA) containing 400 U ml^−1^ penicillin and 400 µg ml^−1^ streptomycin. The tissue was washed twice with phosphate-buffered saline (PBS, Ca^2+^ and Mg^2+^ free, Hyclone, China) and minced finely into small pieces (1 mm^3^) in a new tissue culture dish. The tissue pieces were suspended with 1 ml of MEM complete medium (MEM supplemented with 20% fetal bovine serum (FBS, Life technologies, Carlsbad, CA, USA), 10 ng ml^−1^ bFGF (Invitrogen, Waltham, MA, USA), 100 U ml^−1^ penicillin (HyClone, South Logan, UT, USA), 100 µg ml^−1^ streptomycin (HyClone, South Logan, UT, USA)) and then the tissue suspension was planted into a 25 cm^2^ standard cell culture flasks in succession. The suspension was incubated at 25 °C in a CO_2_ incubator (HF90/HF240; Heal Force, Shanghai, China). After 24 h of attachment, 1 ml MEM complete medium was carefully added to the flasks. Every 4–6 days, half of the medium was removed and replaced with fresh medium until passaging.

When the cells migrating from the muscle tissue pieces stopped growing, the cell clusters were scattered with trypsin digestion (0.25% trypsin and 0.2% EDTA in PBS) and seeded in the original culture flask again. In order to protect the cells’ proliferation activity, upon attaining 80% monolayer, above trypsin digestion was followed to subculture the cells every 5–7 days at 1:1 cell suspension to fresh medium according to the different growth status.

A healthy triploid flounder weighing 131 g was selected and performed on the same day using the similar method of the diploid flounder as mentioned above, but the selected explant (white muscle tissue) was close to the skeleton. In addition, low concentration of trypsin digestion (0.05% trypsin and 0.04% EDTA in PBS) was used in the first passage when the cell clusters were seeded in a new culture flask.

### Cryogenic preservation and recovery

For cryopreservation, the cells at passage 10, 20, 30, 40, 50 and 60 from POMSC_S(2n)_ and POMSC_S(3n)_ were separately suspended in frozen stock solution (MEM complete medium supplemented with 10% dimethhyl sulphoxide (DMSO, Sigma, St. Louis, MO, USA)) at a density of 1–3×10^6^ cells ml^−1^. The cell suspension was dispensed into 2 ml cryogenic vial labeled with the cell name, freezing serial number and date, and kept in a NALGEBE^TM^ Cryo 1 °C Freezing Container (Nalgene, Rochester, NY, USA) at 4 °C for 30 min, −20°C for 2 h, −80°C overnight. Then these vials were transferred into liquid nitrogen (−196 °C) for long-term storage. To recover and reseed the cells, the frozen cells were taken out from liquid nitrogen and quickly thawed in 42 °C water bath. Following removal of the freezing medium by centrifugation (2,200 g, 2 min), cells were transferred into a new flask and resuspended in complete MEM medium, and cultured at 25 °C.

### Growth curves of cells

The cell growth was assessed by counting cell numbers with hemocytometer under a BDS300-PH inverted microscope (Optec, Chongqing, China). Firstly, 9.0×10^4^ cells ml^−1^ at passage 48 of POMSC_S(2n)_ and passage 47 of POMSC_S(3n)_ were severally seeded into 24-well plates and incubated at 25 °C. On day 1, 3, 5, 7, 9 and 11, four wells of POMSC_S(2n)_ and POMSC_S(3n)_ cells were respectively counted using a haemocytometer. Cell growth curves were drawn, and the population doubling time (PDT) was calculated according to the equation PDT = *t*_∗_[lg2/(lg*N_t_* −lg*N_o_*)] (*N*_0_, the number of cells recorded at 24 h after inoculated; *t*, incubation time; *N_t_*, the number of cells recorded at *t* h) (Pan et al., 2012).

### Chromosome analysis

POMSC_S(2n)_ cells at passage 30 and POMSC_S(3n)_ cells at passage 29 were prepared to analyze chromosomal karyotype. Briefly, 1.0×10^6^ cells were separately inoculated into a 25 cm^2^ culture flask and incubated at 25 °C overnight. After 24 h, the cells were subsequently incubated at 25 °C with colchicine (1.0 µg ml^−1^) for 3 h in the same flask, and then the monolayer was trypsinized and harvested by centrifugation (1,000 g, 6 min). The supernatant was discarded and the cells were suspended in 10 ml hypotonic solution of 0.075 mol L^−1^ KCl for 25 min at 37 °C, then prefixed 5 min in 2 ml of cold fresh Carnoy’s fixative (methanol: acetic acid = 3:1) by centrifugation (1,000 g, 6 min). Subsequently, the cell pellets were fixed twice in 5 ml cold Carnoy’s fixative, 15 min for each time. After centrifugation (1,000 g, 6 min), cells were suspended in 0.5 ml cold Carnoy’s fixative. Glass slides were prepared using the conventional drop-splash technique and air-dried. Chromosomes were stained with 10% Giemsa for 10 min. One-hundred photographed cells at metaphase were counted under an Eclipse 80I fluorescence microscope (Nikon, Japan). The chromosomal karyotypes were analyzed according to Levan, Predga & Sandberg (1964).

In the meantime, the nuclear-cytoplasmic ratios of POMSC_S(2n)_ and POMSC_S(3n)_ cells were respectively calculated according to the measurement values of 20 cells under the Eclipse 80I fluorescence microscope.

### Skeletal muscle satellite cell gene marker analysis

The cell types of the two cell lines were verified with analysis of *pax7b* (Jiao et al., 2015a) skeletal muscle satellite cell gene marker. Total RNAs were distinctly extracted from POMSC_S(2n)_ at passage 53 and POMSC_S(3n)_ at passage 52 using RNA isolation kit (TIANGEN, China). The RNAs were incubated with RNase-free DNase I (Promega, Madison, WI, USA) to eliminate contaminating genomic DNA before being reverse-transcribed into cDNA using oligodT primers and M-MLV reverse transcriptase (Promega, Madison, WI, USA) according to the manufacturers’ instructions. PCR was carried out in a volume of 25 µl containing 1 µl (400 ng) of cDNA as template, 0.5 µl of each primer (10 µM), 10.5 µl nuclease-free water and 12.5 µl of 2×*Pfu* MasterMix (CWBIO, Beijing, China). PCR was run as follows: 94 °C for 5 min, 35 cycles of 94 °C for 30 s, 52 °C for 30 s and 72 °C 30 s, and then 72 °C 10 min for elongation. A RT-PCR minus control was also included. The 198bp PCR products were analyzed by 1% agarose gel electrophoresis.

### Immunocytochemical identification

The POMSC_S(2n)_ cells at passage 56 and POMSC_S(3n)_ cells at passage 55 were examined for the expression of Desmin as a myogenic cell marker (Wang & Rudnicki, 2012). About 1.0–1.2 × 10^5^ cells were respectively inoculated in one 24-well plate and incubated at 25 °C for 72 h. Cells were washed three times in cold PBS, fixed in paraformaldehyde (4.0% in PBS, v/v) for 10 min at room temperature, washed for 5 min in cold PBS, perforated 15 min in cold 0.5% Triton X-100 PBS, washed twice in PBS, blocked for 30 min in 1% BSA, and then incubated with the primary antibody (2.5 µl Anti-Desmin Antibody produced in rabbit (D 8281; Sigma, St. Louis, MO, USA) dissolved in 200 µl 1% PBS) at 4 °C overnight. After washed twice in PBS, cells were incubated with the appropriate secondary antibody (2.5 µl Biotin-labeled Goat anti-Rabbit IgG (H + L) antibodies (Invitrogen, Waltham, MA, USA) dissolved in 200 µl 1% PBS) at 25 °C for 1 h. Then washed in PBS once more, and peroxidase activity was visualized with 3, 3′ N-Diaminobenzidine Tertrahydrochloride (DAB; CWBIO, Beijing, China) in Tris–HCl buffer (pH 7.6) containing H_2_O_2_. Labelled cells were examined under the Eclipse 80I fluorescence microscope. Negative controls (without the primary antibody) were included in the experiment.

### Skeletal muscle satellite cell differentiation ability test

POMSC_S(2n)_ cells at passage 32 and POMSC_S(3n)_ cells at passage 33 were individually inoculated into a 12 well plate at a density of 1–2×10^4^ cells/ml in growing medium (GM, MEM mixed with 20% fetal bovine serum (FBS), 10 ng ml^−1^ bFGF, 100 U ml^−1^ penicillin and 100 µg ml^−1^ streptomycin). After 2 days, differentiation induction of cells was separately performed by switching 80–90% confluent cells to differentiation medium (DM) supplemented with 2% horse serum (GIBCO, Waltham, MA, USA), 100 U ml^−1^ penicillin and 100 µg ml^−1^ streptomycin. The medium was changed once every 2 days. Control groups with GM all along experimental period were performed. Cells from induction and control groups were respectively harvested on the 6th day. The 4′, 6-diamidino-2-phenylindole (DAPI, Sigma, St. Louis, MO, USA) was used to stain the cell nucleus. In detail, cells were washed in cold PBS, fixed in paraformaldehyde (4.0% in PBS, v/v) for 10 min at room temperature, washed 5 min in cold PBS, perforated 15 min in cold 0.5% Triton X-100 PBS, washed twice in cold PBS, stained with 0.5 mg/ml DAPI for 2 min. Then, labelled cells were visualized under the Eclipse 80I fluorescence microscope. The differentiation experiment had three replicates.

### Immunocytofluorescence of myosin heavy chain

To confirm that the long fibre-like cells after differentiation are myotubes, cells of POMSC_S(2n)_ and POMSC_S(3n)_ at passage 40 and 39 were respectively inoculated into 12-well plates at a density of 1–1.5×10^5^ cells/ml in growing medium GM. After 48 h, confluent cells were switched into DM. The medium was changed once every 2 days. On the 4th day of differentiation, cells were harvested. Simply, cells were washed in PBS, fixed in paraformaldehyde (4.0% in PBS, v/v), perforated in 0.5% Triton X-100 PBS, blocked in 1% BSA, and then incubated with the primary antibody (2.5 µl Anti- Myosin Heavy Chain Monoclonal Antibody produced in mouse (RLM3058, Ruiying Biological, China) dissolved in 200 µl 1% PBS) at 4 °C overnight. After washed in PBS, cells were incubated with the secondary antibody (1.5 µl Goat anti-Mouse IgG (H + L) Secondary Antibody, Cy3 conjugate (A10521; Thermo Fisher, Waltham, MA, USA) dissolved in 200 µl 1% PBS) at 25 °C for 1.5 h. Then cells were washed in PBS once more and stained with 0.5 mg/ml DAPI for 2 min. Labelled cells were observed under the Eclipse 80I fluorescence microscope. The differentiation experiment had three replicates. Negative controls (without the primary antibody) were included in the experiment.

### Transfection test with pEGFP-N_3_ reporter gene

POMSC_S(2n)_ cells from passage 24 and POMSC_S(3n)_ cells from passage 22 were respectively seeded in a 24-well plate at a density of 4×10^5^ cells well^−1^. Sub-confluent monolayers were separately transfected with pEGFP-N_3_ express vector (Invitrogen, USA) using lipofectamine^TM^ 2000 (Invitrogen, St. Louis, MO, USA). Briefly, 1 µl lipofectamine^TM^ 2000 and 2 µl pEGFP-N_3_ (400 ng µl^−1^) were dissolved respectively in 49 µl and 48 µl of MEM (without FBS or antibiotics). After 5 min, the two solutions were mixed and incubated for 25 min. Subsequently, the mixture was added gently into the well and the cells were cultured for 4 h at 25 °C, then the supernatant was substituted with fresh complete medium immediately. The green fluorescence signals were observed under an Eclipse TE2000-U fluorescence microscope (Nikon, Tokyo, Japan) during the next one week regularly.

### Statistic analysis

Data from POMSC_S(2n)_ and POMSC_S(3n)_ were analyzed with the SPSS package for Windows (Version 15.0, SPSS, Chicago, IL , USA). Significant differences were determined using One-way ANOVA tests followed by Duncan’s multiple comparison tests at the probability level of 0.05.

**Figure 1:**
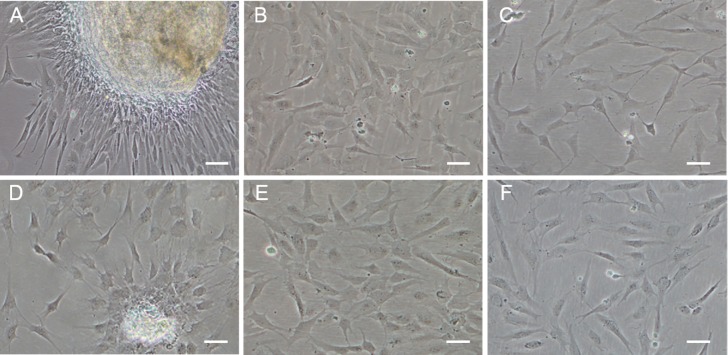
Monolayer of the POMSC_S(2n)_ and POMSC_S(3n)_ cells. POMSC_S(2n)_: (A) primary culture at day 11; (B) passage 27; (C) passage 61. POMSC_S(3n)_: (D) primary culture at day 9; (E) passage 30; (F) passage 60, both appear predominantly spindle-like. *Scale bar* 50 µm.

## Results

### Primary culture and subculture

Approximately 4–7 days after the muscle tissue fragments adhered to the bottom of the 25 cm^2^ culture flask, cells began to migrate from the edges of the seeded tissue samples. Confluent monolayers of cells were observed on the 8–12th day (Figs. 1A and 1D). When these cells grew into colonies, the first subculture was conducted. The cells were subcultured at 1:2 cell suspension to fresh medium every 4–7 days when they reached 90% confluence. In this way, cells were repeatedly passaged, and two cell lines (POMSC_S(2n)_ and POMSC_S(3n)_) from flounder skeletal muscle were established (Figs. 1B–1C, 1E and 1F). To date, the POMSC_S(2n)_ and POMSC_S(3n)_ cell lines have been subcultured for 67 passages and 66 passages, respectively. Morphologically, the cell lines are both composed of mainly spindle cells and a small number of epithelial cells. The nuclear-cytoplasmic ratios are 0.0409 ± 0.0043 for POMSC_S(2n)_ cells at passage 26 and 0.0546 ± 0.0061 for POMSC_S(3n)_ cells at passage 27.

### Cryogenic preservation and recovery

Cells were cryopreserved at different passages in liquid nitrogen (−196 °C). After storage of 3–5 months, the cells were successively thawed and seeded into flasks with survival rates of 90–95% for POMSC_S(2n)_ and 85–90% for POMSC_S(3n)_. Both cells successfully grew to confluence in 5 days. Almost no apparent morphological differences compared with fresh cells were observed.

### Growth curve

**Figure 2:**
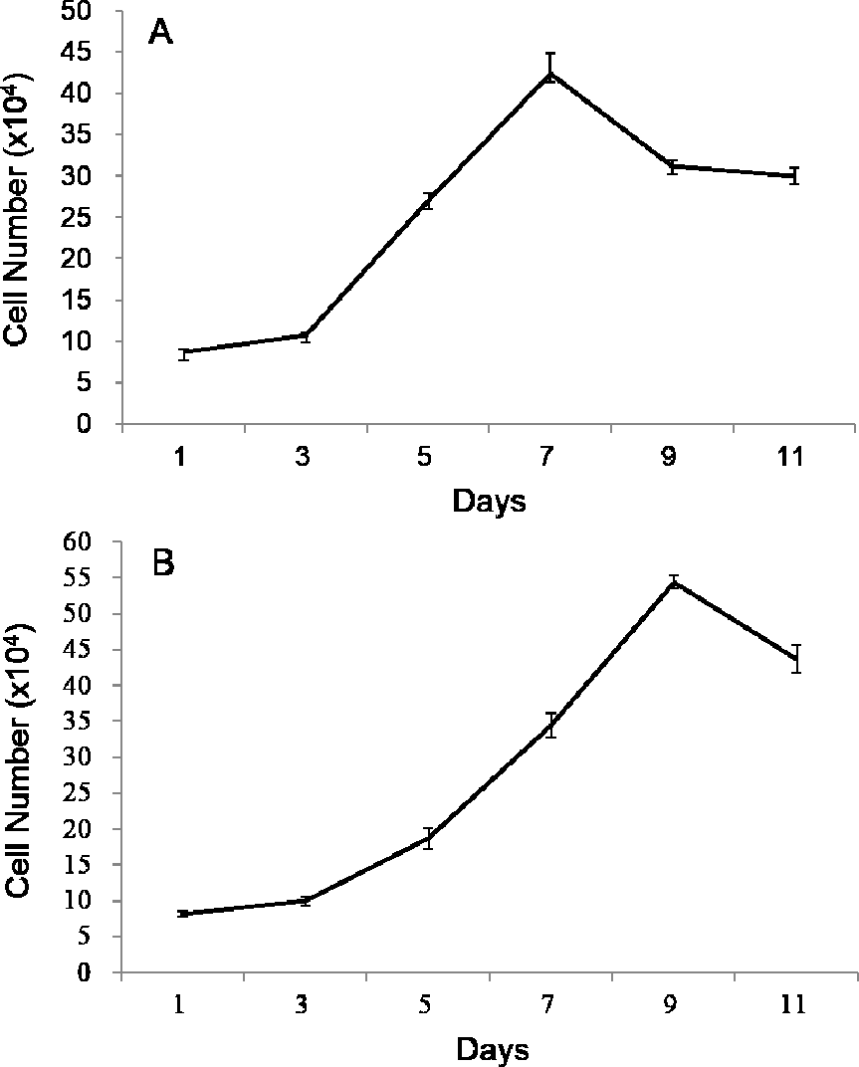
Growth curves of POMSC_S(2n)_ and POMSC_S(3n)_ cells in MEM supplied with 20% FBS at 25 °C. The cell number was counted every other day. The starting cell numbers were distinctly 8.70 × 10^4^ cells for (A) POMSC_S(2n)_ and 8.19 × 10^4^ cells for (B) POMSC_S(3n)_ per well of a 24-well plate at 24 h after seeded. Value are means ± SE (*n* = 4).

Both growth curves of POMSC_S(2n)_ and POMSC_S(3n)_ were different from the typical “S” shape, which all showed a sharp rise and drop in the growth tendency (Fig. 2). The plateau period was absent, and after the exponential growth phase, the cells died successively. According to the exponential growth equation PDT =*t*_∗_[lg2/(lg*N_t_* − lg*N_o_*)], the doubling times were 60.92 ± 2.17 h at a inoculum density of 8.70 × 10^4^ cells ml^−1^ for POMSC_S(2n)_ and 69.88 ± 0.37 h at a inoculum density of 8.19×10^4^ cells ml^−1^ for POMSC_S(3n)_. It revealed that POMSC_S(2n)_ cells proliferated more rapidly than POMSC_S(3n)_ cells (*P* < 0.05) when cultured at the same temperature, media and serum concentrations.

### Chromosomal karyotype

**Figure 3:**
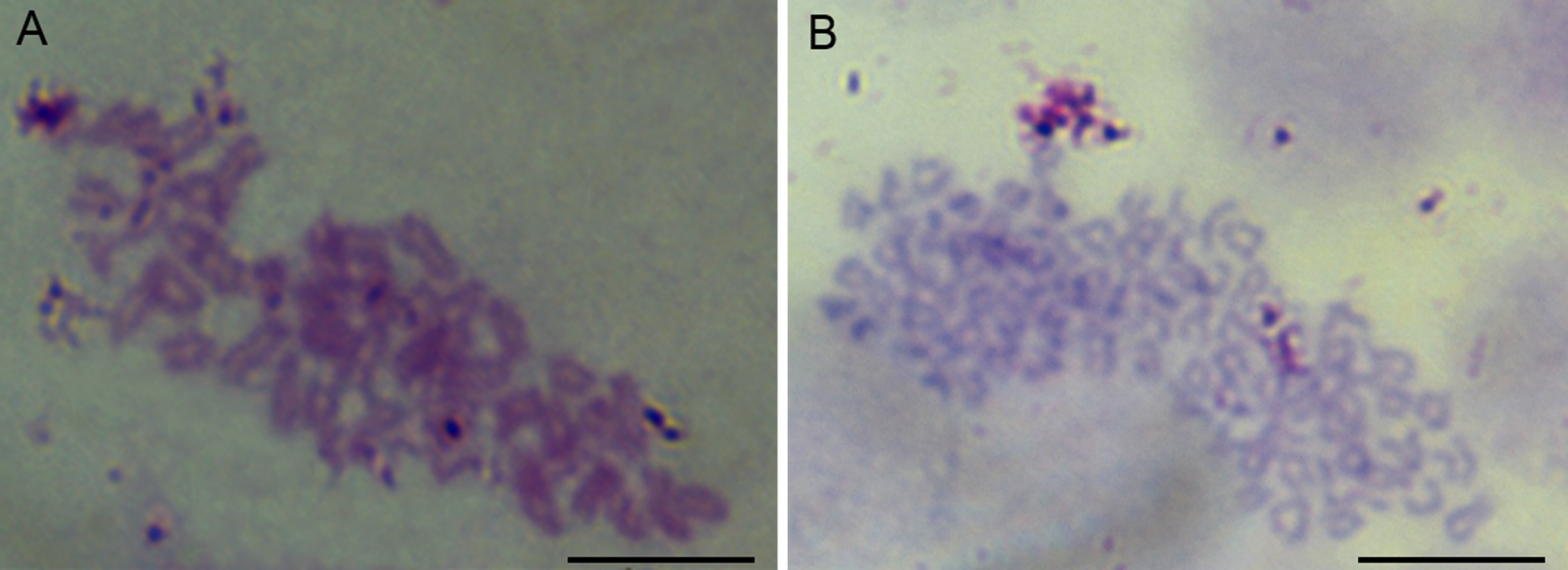
Chromosome metaphases of POMSC_S(2n)_ and POMSC_S(3n)_ cells. Cellular chromosomes of POMSC_S(2n)_ cells at passage 30 (2n = 48, A) and POMSC_S(3n)_ cells at passage 29 (3n = 72, B) arrested in metaphase. One hundred metaphases were counted, respectively. *Scale bar* 10 µm

The chromosome assays showed that the chromosomes of cells were all telocentrics (Fig. 3). The modal numbers of POMSC_S(2n)_ and POMSC_S(3n)_ are 48 and 72 counted at passage 30 or passage 29, respectively. The metaphases both displayed normal karyotype morphology, and their chromosomal karyotypes are 2n = 48*t* and 3n = 72*t*.

### Skeletal satellite cell gene marker identification

**Figure 4:**
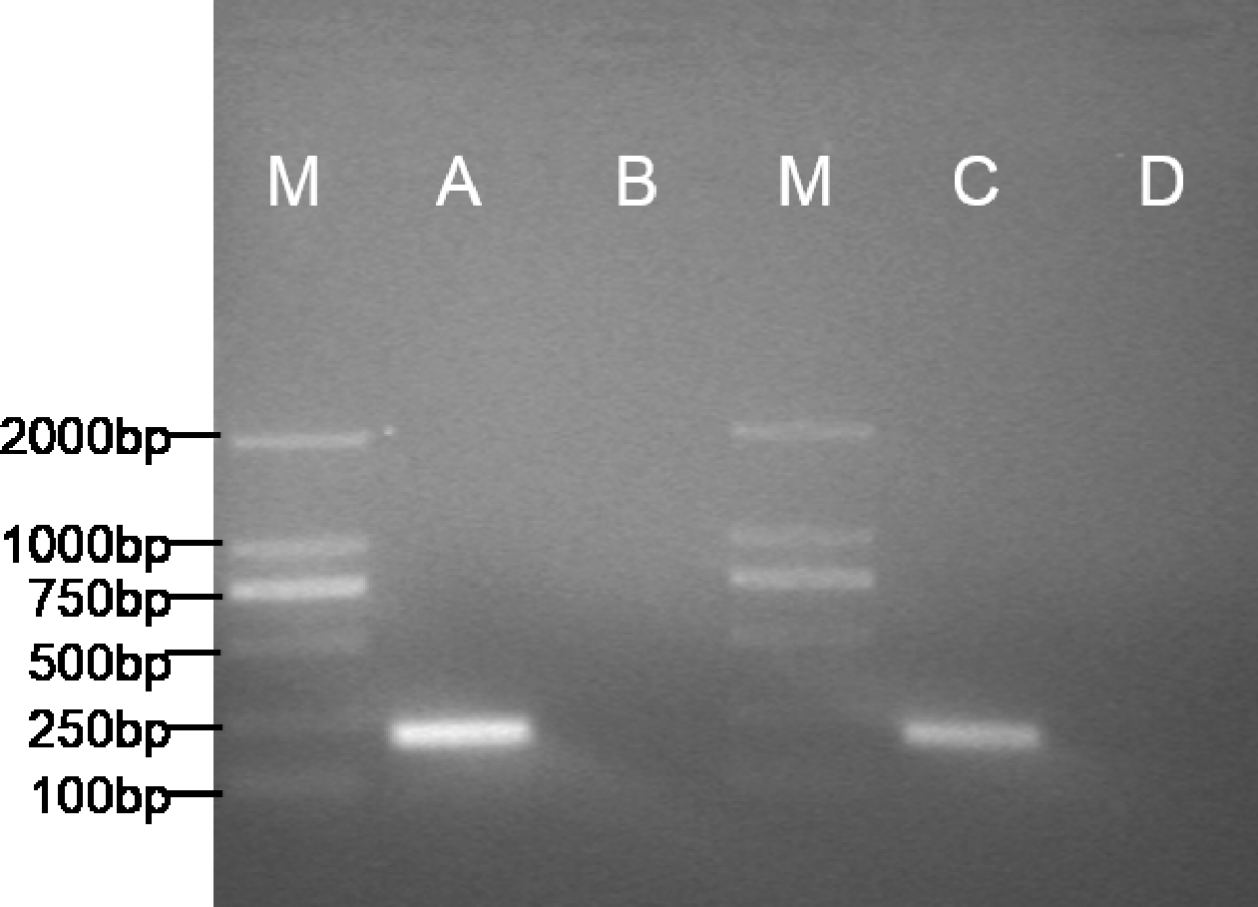
Specific expression pattern of *pax7b* gene in POMSC_S(2n)_ and POMSC_S(3n)_ cells. M: DS™2000 Marker; (A) expression of *pax7b* in POMSC_S(3n)_ cells; (B) minus control for POMSC_S(3n)_ cells; (C) expression of *pax7b* in POMSC_S(2n)_ cells; (D) minus control for POMSC_S(2n)_ cells.

The expected 198bp fragment of *pax7b* (Fig. 4) was respectively amplified from the POMSC_S(2n)_ cells at passage 53 and POMSC_S(3n)_ cells at passage 52. Sequencing results showed 100% match with the known flounder gene sequence. The types of these two cell lines should be skeletal muscle satellite cells.

### Immunocytochemical characterization

**Figure 5:**
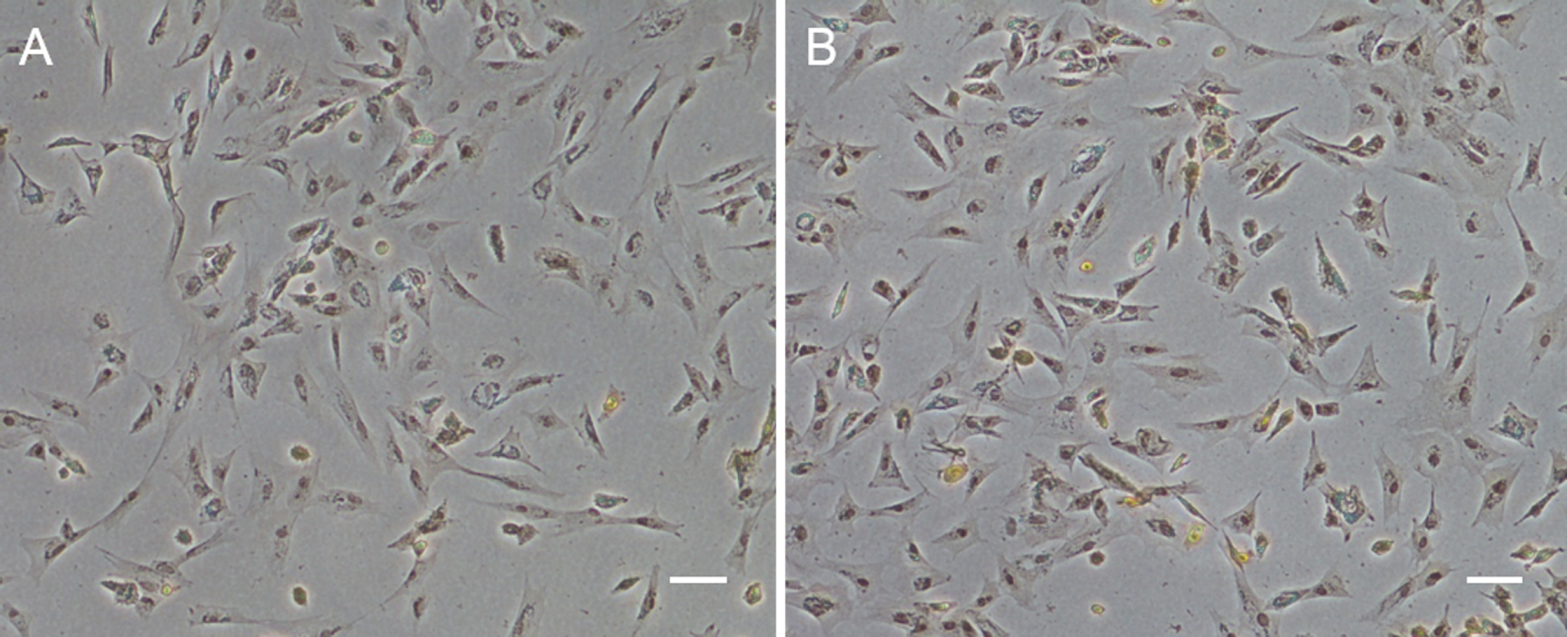
Cell immunohistochemistry microscopy images. POMSC_S(2n)_ and POMSC_S(3n)_ cells were characterized by expression of myogenic cell marker, Desmin, with intensity of the signal varying among cells. (A) expression of Desmin in POMSC_S(2n)_ cells; (B) expression of Desmin in POMSC_S(3n)_ cells. *Scale bar* 50 µm.

The cells of POMSC_S(2n)_ and POMSC_S(3n)_ were respectively labeled by Desmin antibodies, although the intensity of the labelling varied (Fig. 5). It proved that the cells of POMSC_S(2n)_ and POMSC_S(3n)_ should be myogenic cells.

### Identification of differentiation ability

**Figure 6:**
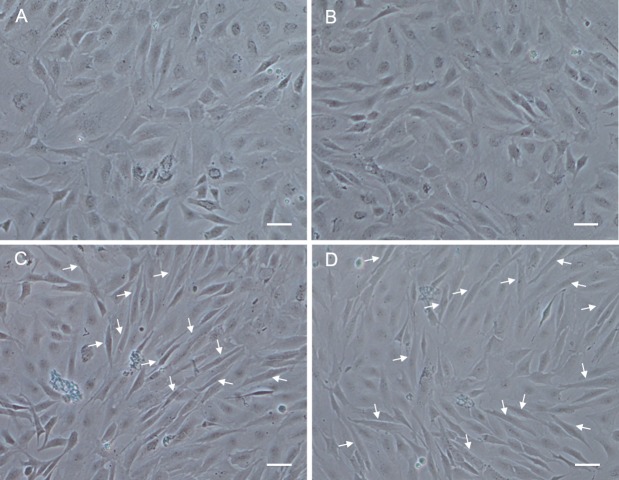
Morphological changes of POMSC_S(2n)_ and POMSC_S(3n)_ cells after differentiation. At 48 h after changed to DM, long fibre-like cells (arrows) could be observed both in the POMSC_S(2n)_ and POMSC_S(3n)_ compared with the controls. (A) control of POMSC_S(2n)_; (B) control of POMSC_S(3n)_; (C) differentiation of POMSC_S(2n)_; (D) POMSC_S(3n)_ differentiation of. Scale bar 50 µm.

**Figure 7:**
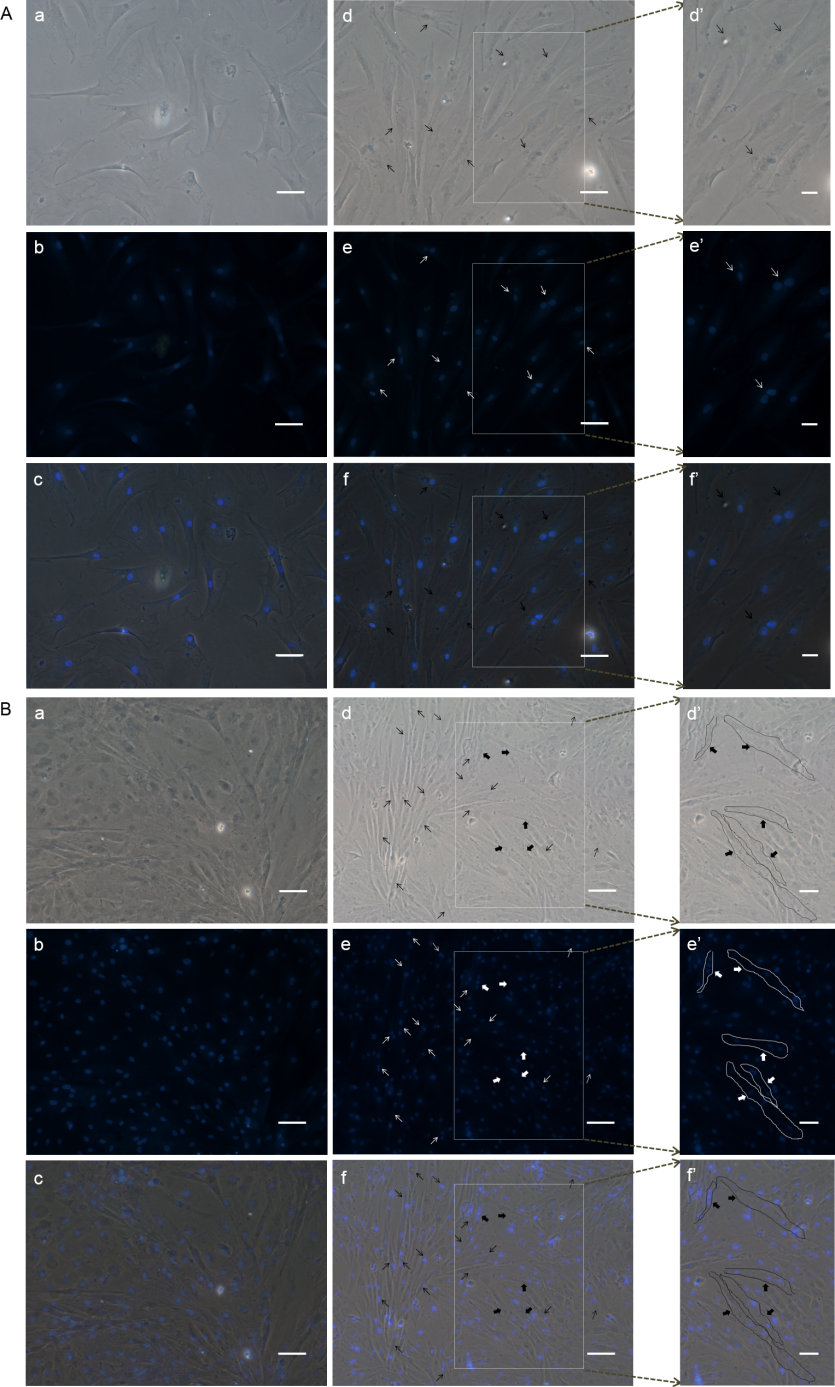
DAPI staining to check the differentiation of POMSC_S(2n)_ and POMSC_S(3n)_ cells. Certain numbers of multinucleated myotubes with about 2–4 nuclei per cell were respectively observed from the differentiation groups of POMSC_S(2n)_ and POMSC_S(3n)_ at 96 h after switched into DM. *Arrows* (*black* or *white*) indicate fibroid cells of double nucleus almost found only in differentiation groups and *solid arrows* (*black* or *white*) showed cells with 3 or 4 nuclei per cell.

In order to analyze the differentiation ability of POMSC_S(2n)_ and POMSC_S(3n)_, cells were plated at a low density firstly in GM, then switched to DM. At 48 h after changed to DM, long fibrous cells could be observed in POMSC_S(2n)_ and POMSC_S(3n)_ compared with the controls (Fig. 6). The transition from a proliferating to a differentiation phenotype was further checked by DAPI staining. Certain numbers of multinucleated myotubes with about 2 or 3–4 nuclei per cell were respectively observed in the treatment groups of POMSC_S(2n)_ or POMSC_S(3n)_ but the control groups (Figs. 7A and 7B). The results showed that both POMSC_S(2n)_ and POMSC_S(3n)_ cells have the differentiation abilities to differentiate into small multinucleated myosatellite fibres.

### Differentiation index detection

**Figure 8:**
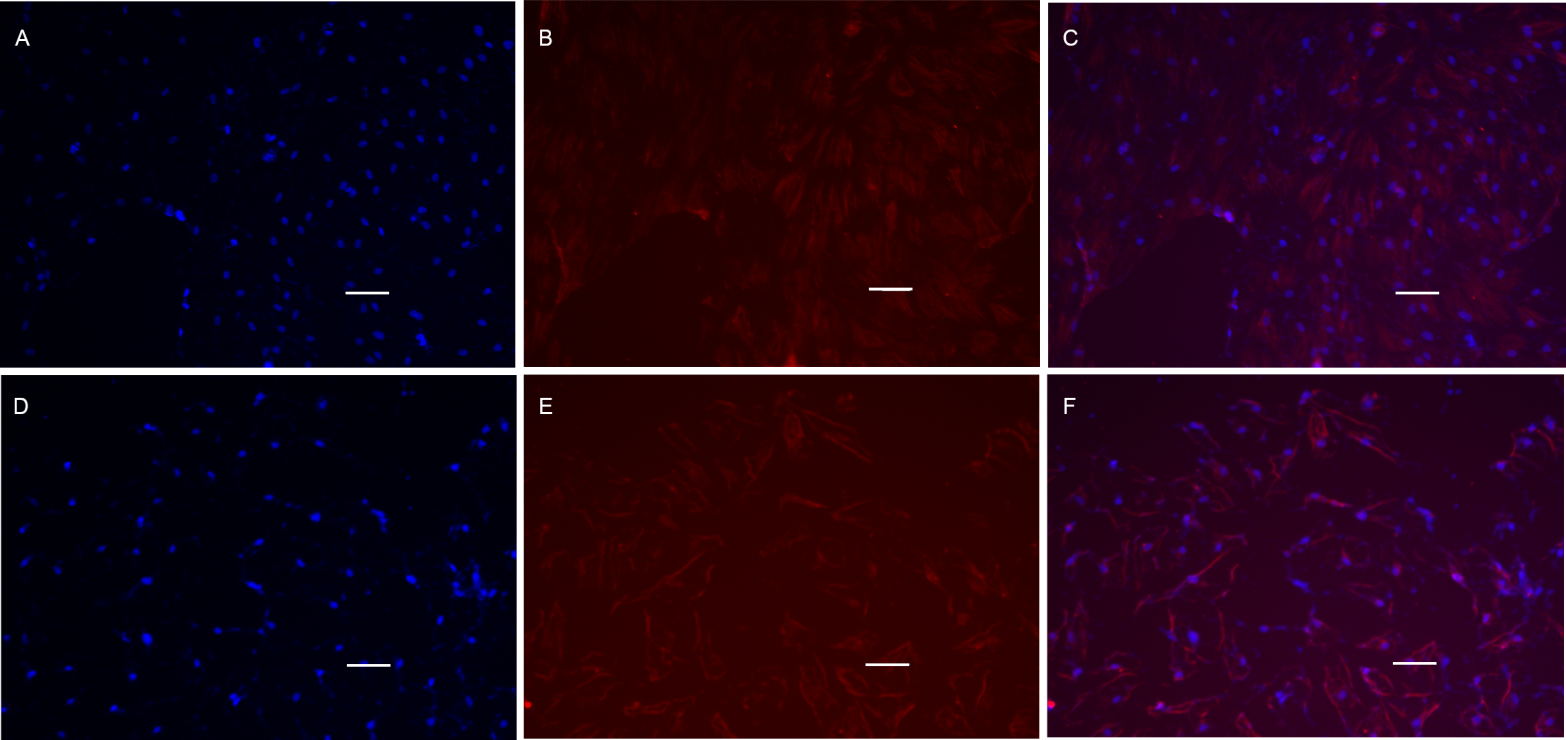
Cell immunofluorescence microscopy images of MyHC protein. POMSC_S(2n)_ and POMSC_S(3n)_ cells were characterized by expression of MyHC (red) protein on the 4th day of differentiation, with intensity of the labelling varying among cells. Nuclei were stained with DAPI (blue). POMSC_S(2n)_: (A) DAPI staining; (B) fluorescent image; (C) overlap; POMSC_S(3n)_: (D) DAPI staining; (E) fluorescent image; F, overlap. *Scale bar* 25 µm.

POMSC_S(2n)_ and POMSC_S(3n)_ cells were first cultivated in GM for 2 days, then switched to DM. On the 4th day of differentiation, anti-myosin heavy chain antibody staining was performed to demonstrate the expression of sarcomeric myosin proteins. Cells of POMSC_S(2n)_ and POMSC_S(3n)_ both displayed positive of myosin heavy chain (MyHC) (Fig. 8), which was considered as the differentiation index. The results clearly showed that the multinucleated myosatellite fibres after differentiation should be myotubes.

### Transfection efficiency analysis

**Figure 9:**
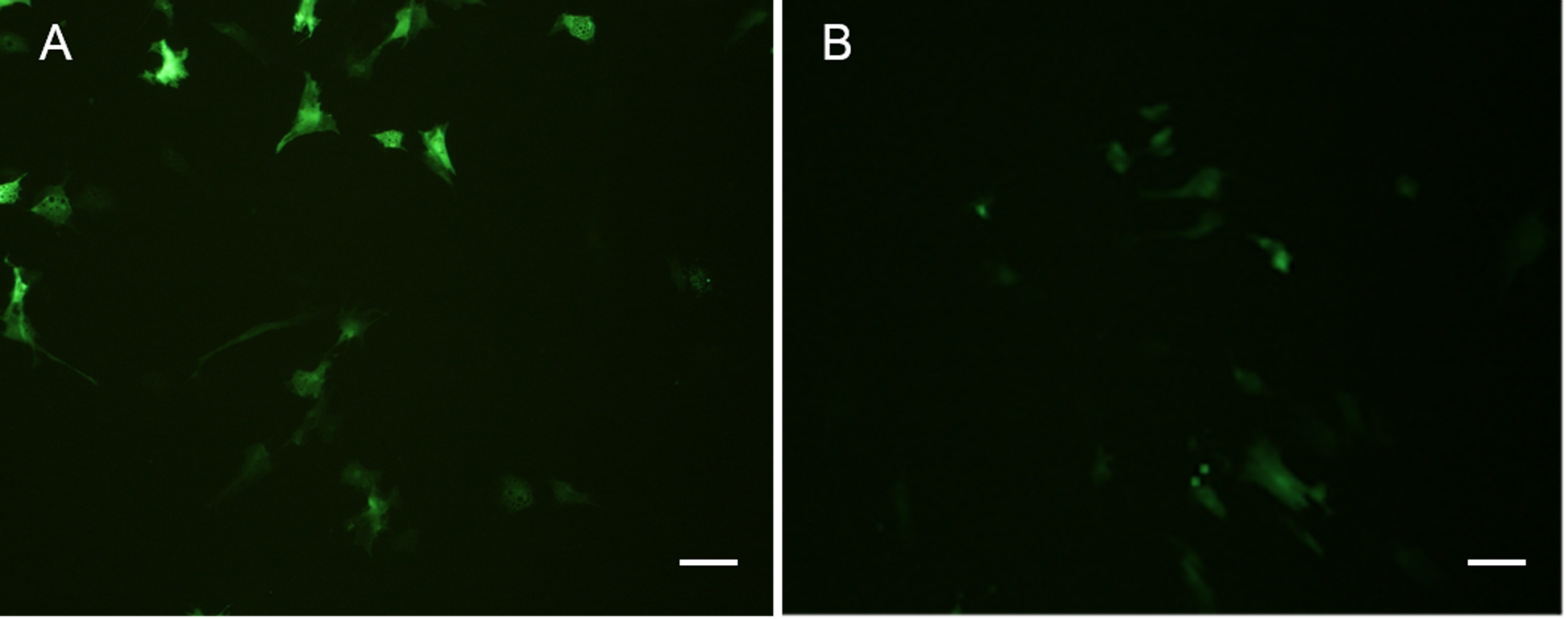
Green fluorescent protein (GFP) expression in transfected POMSC_S(2n)_ cells and POMSC_S(3n)_ cells. Green fluorescent protein (GFP) expression in transfected POMSC_S(2n)_ cells at passage 24 (A) and POMSC_S(3n)_ cells at passage 22 (B) respectively transfected with pEGFP-N3. *Scale bar* 50 µm.

Both POMSC_S(2n)_ and POMSC_S(3n)_ cells were successfully transfected with pEGFP-N_3_ reporter gene, and the expression of EGFP in these cells could be detected after 16 h (Fig. 9). The numbers of green fluorescence signals increased gradually during the next few days until the 8th day. The transfection efficiencies were found to be 20–30% in POMSC_S(2n)_ cells and15–25% in POMSC_S(3n)_ cells, respectively. Results indicated the suitability of POMSC_S(2n)_ and POMSC_S(3n)_ for transfection.

## Discussion

**The culture condition selection of POMSCs *in vitro***

The method of explant culture was used in this paper, so far, the cells from POMSC_S(2n)_ and POMSC_S(3n)_ were respectively subcultured for 67 passages and 66 passages. The primary culture protocol of MSCs similar to the mammals was carried out in gilthead sea bream and trout (Montserrat et al., 2007; Gabillard, Sabin & Paboeuf, 2010). The MSCs are commonly separated and purified from whole muscle by a process including mincing, a series of enzymatic digestions and differential adhesion time method or incontinuous density percoll gradient centrifugation technique (Yang et al., 2004; Jiao et al., 2011). However, this process could affect the cell viability and a varying small proportion of the muscle cells could be obtained. It is also needed to treat the plastic or glass culture flask bottom with poly-lysine and laminin. So the operational procedure is tedious, easily contaminated and arduous operation (Rosenblatt et al., 1995; Zhang et al., 2006a; Li et al., 2010). In view of the result that MSCs from flounders *in vitro* could adhere and proliferate well on the culture flask without aforementioned promoting anchor materials, we think that the primary method of explant culture could overcome the aforementioned disadvantages, and the cell viability could be protected sufficiently (Luo et al., 2006; Chen et al., 2011).

MEM supplemented with 20% FBS and bFGF was used for the proliferation of POMSCs in the present study. Some of these cells could differentiate into the large polynucleated myocyte cells after they were transferred into MEM supplemented with 2% HS. Generally, the culture media are different along with the proliferation or differentiation culture of MSCs derived from different species. A high concentration of serum could promote the proliferation of myosatellite cells, while a low concentration of serum would promote the differentiation of myosatellite cells to myotubes. Basic fibroblast growth factor (FGF2 or bFGF) could stimulate cell proliferation of MSC_S_ (Li et al., 2010). In particularly, DMEM supplemented with 15% horse serum and McCoy’s 5A supplemented with 15% chicken serum were selected in the proliferation of cells derived from the rat, sheep, cattle and chicken, respectively. A proliferation medium (F10 + 10%FCS) and a differentiation medium (DMEM + 2%FCS) were used in either the proliferation or the differentiation of trout satellite cells cultured *in vitro*Gabillard, Sabin & Paboeuf (2010). Rat skeletal muscle satellite cells were also observed to differentiate into myotubes along with the decrease of bFGF (Allen et al., 1985). According to above reports and our results, we believe that the myosatellite cells from flounder had a better adaptability to the culture conditions *in vitro*, and the growth of these cells only needs a common culture condition that the myosatellite demanded. Our results also showed that a high concentration of bFGF in medium is needed for the proliferation and undifferentiation of myogenic satellite cells *in vitro*, what similar results were also reported by Allen et al. (1985). It is contrary to the understanding about bFGF *in vivo* that it could awaken the differentiation of myosatellite cells, activate the myoblast and promote the regeneration of the damaged tissues (Su et al., 2004; Li et al., 2010).

### Characters of POMSCs

In this paper, the myosatellite cells that isolated from flounder could proliferate in MEM medium continuously, and the morphology of mononuclear cells was maintained in most of the cells. The growth curves of POMSC_S(2n)_ and POMSC_S(3n)_ both showed a steep rise and down, which were different from the typical “S” shape. The specific reason is not clear at present. Differentiation experiment proved that the cells were capable of differentiating into the multinucleated cells *in vitro* which was the basis of myotube and muscle fibre. The myosatellite cells become increasingly diluted with the age of fishes, but as the crucial basis of the muscle hyperplasic and hypertrophic growth, the total number of myosatellite cells in the white axial muscle might be rather constant during the growth phase of the fish (Koumans et al., 1992). Wang & Rudnicki (2012) mentioned that because satellite cells are self-renewing, within the heterogeneity there must be a stem cell-like population existed, that is resistant to differentiation and maintain the satellite cell pool, meanwhile, there must be a committed satellite cell population existed, which could differentiate into the myocytes and myotubes. Two main types of cells, mononuclear cells in GM and multinucleated cells in DM were observed in POMSC_S(2n)_ and POMSC_S(3n)_. And the cells of POMSC_S(2n)_ and POMSC_S(3n)_ after differentiation displayed positive to MyHC, which was considered as the differentiation index (Gabillard, Sabin & Paboeuf, 2010). Then, combined with the expression results of *pax7b* and Desmin, we imply that POMSCs could be a mixture population of satellite stem cells and committed satellite cells of skeletal muscle. Further study is needed to confirm this.

The resulting variation in muscle cellularity is thought to be significant determinants of texture and other flesh quality characteristics in aquaculture (Johnston, 1999; Zhu et al., 2014), but there are few reports about the theoretical and applied research of myosatellite cells in teleost, especially in marine fish. The only study was reported by Mommsen (2001) who used the primary culture of muscle cells of gilthead sea bream to examine insulin-like growth factor-I (IGF-I) binding and receptor signal transduction.

### Comparison of MSC_S_ from diploid and triploid flounder

Our results exhibited different characters between triploid and diploid myosatellite cells. The nuclear-cytoplasmic ratio of POMSC_S(3n)_ cells was 1.33 times of POMSC_S(2n)_ as expected. The proliferation rate of POMSC_S(3n)_ was slightly slower than POMSC_S(2n)_. While POMSC_S(3n)_ had stronger differentiation capacity than POMSC_S(2n)s_. In the differentiation experiment, more multinucleated cells, especially myotubes with 3–4 nuclei, could be observed in POMSC_S(3n)_ than POMSC_S(2n)_.

MSC_S_, on activation, participate in myogenic function and provide the biological basis for longitudinal and cross-sectional growth (Dayanidhi & Lieber, 2014). In the light of available literature data, the nucleus constitutes of a significant proportion of the cell volume in muscle fibres at very small fibre sizes. As a result, the relatively large nucleus in triploid contributes to the increased hyperplastic fiber size probably (Suresh & Sheehan, 1998). Meanwhile, Koumans et al. (1993) reported that hyperplasia plays a major role in fish muscle growth. Combing with our results, we speculate that the larger cell volume and the higher differentiation capacity of myosatellite cells contribute to the fast growth of triploid individuals. Further researches should be developed to prove it. Based on the above difference about the growth performance of MSCS between the diploid and triploid, it is worthy to believe that the myosatellite cells from triploid cultured *in vitro* would be helpful to discover the mechanism of triploid muscle growth in the follow-up work. Furthermore, as the triploid fishes have specific physiological features, the myosatellite cells of which could play a key role in the research of biochemistry and molecular evolutionary process of higher vertebrates (Tiwary, Kirubagaran & Ray, 2004).

## Conclusion

Two myosatellite cell lines *in vitro* from diploid and triploid flounder were established, respectively. These heterogeneity cells were mostly spindle-like mononuclear cells. We firstly reported that the myosatellite cells from POMSC_S(2n)_ showed a higher proliferation rate than POMSC_S(3n)_, and POMSC_S(3n)_ had a stronger differentiation capacity than POMSC_S(2n)_. These results could provide a new understanding on the cytological mechanism of muscle growth and development in diploid and polyploid fish species in flounder and other marine fishes. The two cell lines will become the useful tools in the researches concerning the gene function analysis, environment toxicology, pharmacology and so on.

## Supplemental Information

10.7717/peerj.1519/supp-1 Supplemental Information 1 The raw data of the cell counting in the growth curve of cells The resulting of the cell counting that drawing the growth curve of cells.

## Additional Information and Declarations

### Data Availability

The following information was supplied regarding data availability: Raw data is available in the Supplemental Information.
