# Supplementary figures and images for: Comparison of growth characteristics between skeletal muscle satellite cell lines from diploid and triploid olive flounder Paralichthys olivaceus
Source: PeerJ. 2016 Jan 5;4:e1519. doi: 10.7717/peerj.1519 (PMC4715439; doi:10.7717/peerj.1519)

|          | 1.00 | 2.00  | 3.00  | 4.00  | 5.00  | 6.00  |      |
|----------|------|-------|-------|-------|-------|-------|------|
| 2n-肌肉    | 1    | 3     | 5     | 7     | 9     | 11    | x104 |
| ①        | 8.60 | 10.80 | 28.00 | 45.00 | 31.00 | 29.80 |      |
| ②        | 8.50 | 10.50 | 26.60 | 42.00 | 32.00 | 31.00 |      |
| ③        | 9.00 | 11.00 | 26.40 | 40.00 | 30.50 | 29.00 |      |
| average  | 8.70 | 10.77 | 27.00 | 42.33 | 31.17 | 29.93 |      |
| BC=STDEV | 0.26 | 0.25  | 0.87  | 2.52  | 0.76  | 1.01  |      |

|          | 1.00 | 2.00  | 3.00  | 4.00  | 5.00  | 6.00  |      |
|----------|------|-------|-------|-------|-------|-------|------|
| 3n-肌肉    | 1    | 3     | 5     | 7     | 9     | 11    | x104 |
| ①        | 8.64 | 9.51  | 18.92 | 36.23 | 54.59 | 44.78 |      |
| ②        | 8.14 | 9.77  | 20.03 | 34.07 | 55.28 | 44.88 |      |
| ③        | 7.79 | 10.66 | 17.09 | 32.99 | 53.45 | 41.50 |      |
| average  | 8.19 | 9.98  | 18.68 | 34.43 | 54.44 | 43.72 |      |
| BC=STDEV | 0.43 | 0.60  | 1.48  | 1.65  | 0.92  | 1.92  |      |

|          |    | 0    | 1    | 3     | 5     | 7     | 9     | 11    |
|----------|----|------|------|-------|-------|-------|-------|-------|
| average  | 2n | 9.02 | 8.70 | 10.77 | 27.00 | 42.33 | 31.17 | 29.93 |
|          | 3n | 9.02 | 8.19 | 9.98  | 18.68 | 34.43 | 54.44 | 43.72 |
| BC=STDEV |    | 0.11 | 0.26 | 0.25  | 0.87  | 2.52  | 0.76  | 1.01  |
|          |    | 0.20 | 0.43 | 0.60  | 1.48  | 1.65  | 0.92  | 1.92  |

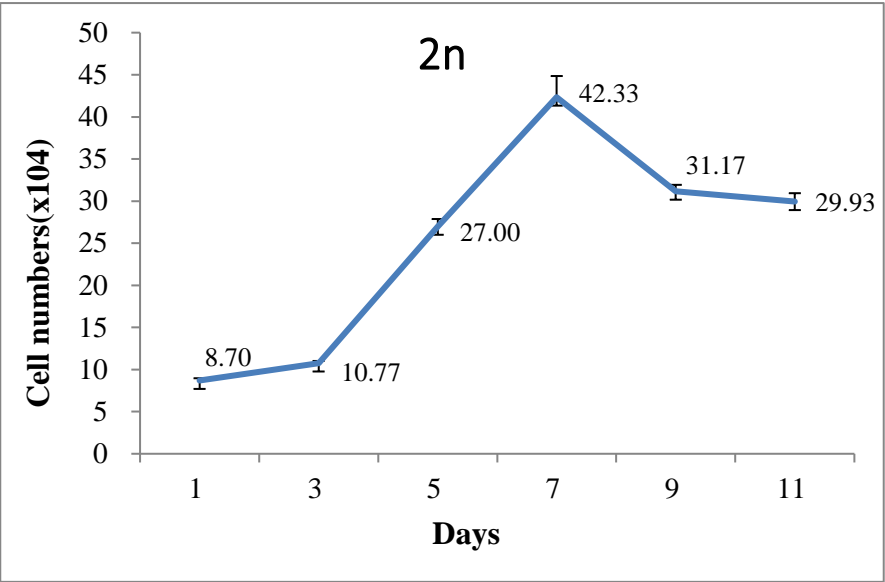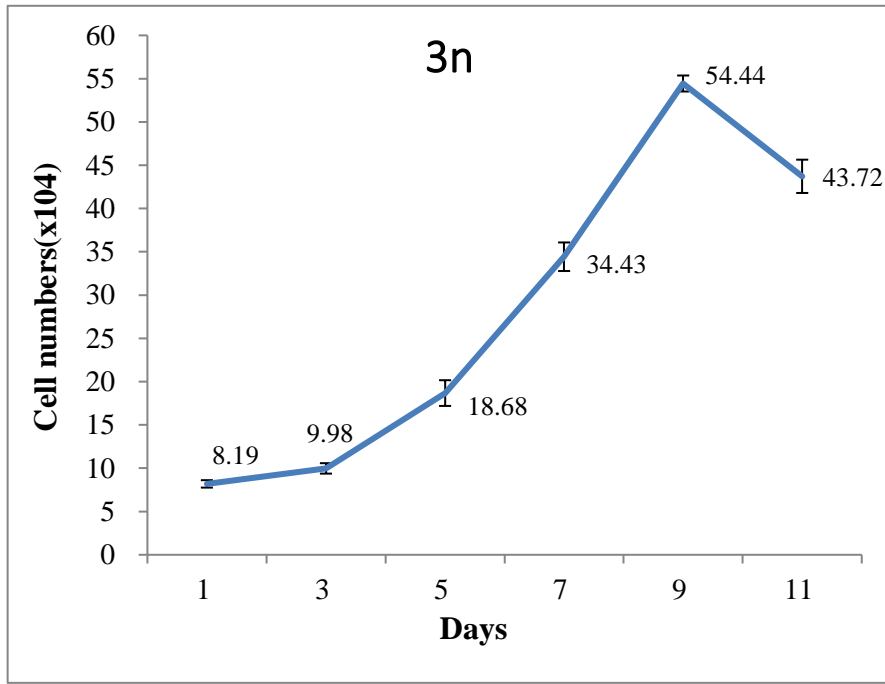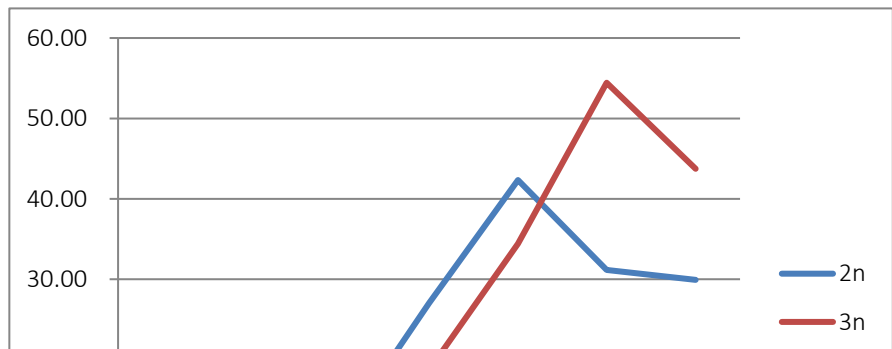

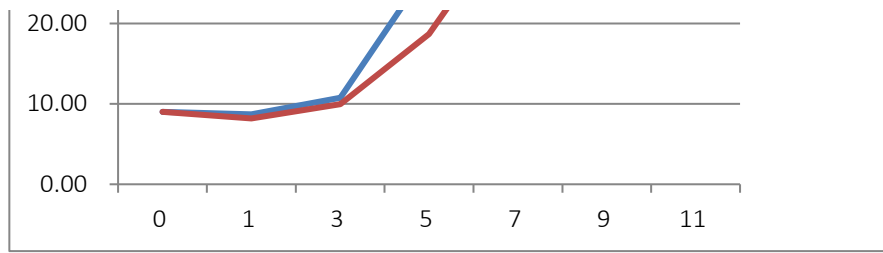

Supplement: Supplemental Information 1 — The resulting of the cell counting that drawing the growth curve of cells. [file peerj-04-1519-s001.pdf]
